# High Incidence of Pathogenic *Streptococcus agalactiae* ST485 Strain in Pregnant/Puerperal Women and Isolation of Hyper-Virulent Human CC67 Strain

**DOI:** 10.3389/fmicb.2018.00050

**Published:** 2018-02-06

**Authors:** Liping Li, Rui Wang, Yan Huang, Ting Huang, Fuguang Luo, Weiyi Huang, Xiuying Yang, Aiying Lei, Ming Chen, Xi Gan

**Affiliations:** ^1^Guangxi Key Laboratory for Aquatic Genetic Breeding and Healthy Aquaculture, Guangxi Institute of Fisheries, Nanning, China; ^2^Bacteria Laboratory, Guangxi Center for Disease Control and Prevention, Nanning, China; ^3^Institute of Animal Science and Technology, Guangxi University, Nanning, China; ^4^Liuzhou's Aquaculture Technology Extending Station, Liuzhou, China; ^5^School of Public Health, National Medical College of Right Rivers, Baise, China

**Keywords:** group B streptococcus, epidemiology, clonal complex (CC), Tilapia, ST485

## Abstract

Group B streptococcus (GBS) is the major pathogen causing diseases in neonates, pregnant/puerperal women, cows and fish. Recent studies have shown that GBS may be infectious across hosts and some fish GBS strain might originate from human. The purpose of this study is to investigate the genetic relationship of CC103 strains that recently emerged in cows and humans, and explore the pathogenicity of clinical GBS isolates from human to tilapia. Ninety-two pathogenic GBS isolates were identified from 19 patients with different diseases and their evolution and pathogenicity to tilapia were analyzed. The multilocus sequence typing revealed that clonal complex (CC) 103 strain was isolated from 21.74% (20/92) of patients and ST485 strain was from 14.13% (13/92) patients with multiple diseases including neonates. Genomic evolution analysis showed that both bovine and human CC103 strains alternately form independent evolutionary branches. Three CC67 isolates carried gbs2018-C gene and formed one evolutionary branch with ST61 and ST67 strains that specifically infect dairy cows. Studies of interspecies transmission to tilapia found that 21/92 (22.83%) isolates including all ST23 isolates were highly pathogenic to tilapia and demonstrated that streptococci could break through the blood-brain barrier into brain tissue. In conclusions, CC103 strains are highly prevalent among pathogenic GBS from humans and have evolved into the highly pathogenic ST485 strains specifically infecting humans. The CC67 strains isolated from cows are able to infect humans through evolutionary events of acquiring CC17-specific type C gbs2018 gene and others. Human-derived ST23 pathogenic GBS strains are highly pathogenic to tilapia.

## Introduction

Group B streptococcus (GBS) is a common species in microbiota of human intestine and vagina (Elliott et al., 1990) and also one of the most important pathogens infecting neonates, genitourinary tract of pregnant/puerperal women and mastitis of dairy cows (Keefe, 1997; Schuchat, 1999; Trijbels-Smeulders et al., 2004). Because of the close relationship between humans and dairy cows, there has been a great concern about the presence of cross-infection between humans and cows (Zadoks et al., 2011). Studies have shown that GBS endangers human health either through direct transmission between cows and humans (Brglez et al., 1979; Hillerton et al., 2004; Manning, 2012) or through evolution of human pathogenic strains from a bovine reservoir (Bisharat et al., 2004). Conversely, others have suggested that humans may act as a source of infection for cows (Dogan et al., 2005; Zadoks and Schukken, 2006), dogs, cats and crocodiles (Yildirim, 2002; Bishop, 2007). Subsequent MLST analysis found strains that are primarily associated with humans but have been reported from cows including members of CC1, ST8, CC19, ST23, and CC26 (Brochet et al., 2006; Sørensen et al., 2010; Zadoks et al., 2011; Manning, 2012). Particularly noteworthy, it was suggested that the hyperinvasive human neonatal clone ST17 is arisen from bovine ST67 (Bisharat et al., 2004). Although a subsequent study did not support that human CC17 is directly from bovine CC67, it did not rule out that CC67 is one of the ancestors involved in CC17 evolution (Sørensen et al., 2010). In last decade, studies found that ST103 strain which was barely detected in dairy cows before has become the dominant ST in dairy cows in Europe and part of East Asia (Zadoks et al., 2011; Yang et al., 2013). Meanwhile, the detection rate of ST485 (DLV of ST103), the almost undetectable ST in human before, had increased significantly to 2.3–7.1% in China (Bohnsack et al., 2008; Lartigue et al., 2009; Manning et al., 2009; Lu et al., 2014, 2016; Wang et al., 2015; Jiang et al., 2016). Therefore, it is worth to study in-depth the causes for sudden significant increase in proportion of CC103 in both dairy cows and human at the same period.

In addition to infecting human and dairy cows, GBS is the main pathogen of fish streptococcus diseases (Bowater et al., 2012; Chen et al., 2012). In recent years, fish *S. agaliae* diseases, especially tilapia *S. agaliae* diseases, are prevalent in the world (Godoy et al., 2013; Li et al., 2013). Studies have demonstrated that fish CC552 strains with serotype Ib are under reductive evolution to a fish-specific CC (Godoy et al., 2013; Rosinski-Chupin et al., 2013). Meanwhile, genome sequence of the highly prevalent ST7 strains with serotype Ia is highly homologous to that of human ST7 strain A909, which also has a strong virulence to tilapia. Therefore, it was suspected that fish ST7 strain might originate from human GBS (Evans et al., 2009; Liu et al., 2013). Our previous study also confirmed that human or cow CC19, CC23, and CC103 containing strains with serotypes Ia, III, and V could infect tilapia and induce clinical signs under experimental conditions (Chen et al., 2015). Furthermore, three independent research groups including ours have found that human GBS is more pathogenic to tilapia than bovine GBS (Garcia et al., 2008; Evans et al., 2009; Pereira et al., 2010; Chen et al., 2015). But up to now, the pathogenicity of human invasive GBS to fish has not been systematically studied.

Based on the close relationship between human and cow GBS as well as fish GBS, we performed molecular epidemiological analysis of GBS with pathogenicity to pregnant/puerperal women and newborns, explored whether CC103 strains are significantly increased in GBS with pathogenicity to pregnant/puerperal women and newborns, and investigated the genetic relationship of CC103 strains that recently emerged in cows and humans through whole genome sequence (WGS) analysis. Meanwhile, we also examined the pathogenicity of 92 pathogenic human GBS to tilapia, and systematically evaluated the risk of cross-host or co-infection between human and fish.

## Methods

### Ethics statement

This study was approved by the Research Ethics Committee of the six hospitals (The People's Hospital of Guangxi Zhuang Autonomous Region, The First Affiliated Hospital of Guangxi Medical University, Maternal and Child Health Hospital of Guangxi Zhuang Autonomous Region, Maternal and Child Health Hospital of Nanning, Maternal and Child Health Hospital of Liuzhou, Affiliated Hospital of Youjiang Medical University for Nationalities), Guangxi Zhuang Autonomous Region, China. All subjects provided written informed consent before their inclusion in the study. All fish infection experiments were conducted according to the principles and procedures of Guangxi Medical University Animal Ethics Committee.

### Invasive GBS isolates

Ninety-two invasive GBS isolates were obtained from 92 patients in 6 hospitals of 3 cities in Guangxi Province. The criteria for selection of the 92 GBS isolates were (1) patients showed typical symptoms of the disease listed in Table 1 and (2) GBS is the absolute dominant group of bacteria isolated from culture source. Among the 92 isolates (Table S1), 72 were from infected pregnant/puerperal women, 6 from infected newborns, and 14 from other infected patients. The isolates were collected from March 2014 to June 2015. Bacterial species were identified using API 20 Strep system (BioMerieux, France) in accordance with the manufacturer's instructions. All isolates were further identified using specific PCR as reported previously (Chen et al., 2012).

**Table 1:** Distribution of 92 invasive GBS strains by isolate source and age of patients^a^.

**Disease of patient**	**Strain Number(%)**	**Patient sex (No.)**	**Patient age/year**	**Culture source (No.)**
Vaginitis	21 (22.83)	Female	19–53	Vaginal secretions (21)
Threatened abortion	15 (16.30)	Female	23–36	Amniotic fluid (3); Cervical secretions (12)
Premature rupture of membranes^b^	14 (15.22)	Female	23–41	Amniotic fluid (12); Cervical secretions (2)
Cervicitis	9 (9.78)	Female	20–46	Cervical secretions (9)
Urethritis	8 (8.70)	Male (4), Female (4)	4–47	Urine (4); Urethral secretions (4)
Pelvic inflammatory disease	6 (6.52)	Female	23–42	Cervical secretions (6)
EOD^c^	4 (4.35)	Male (2), Female (2)	6 h−6 d	Blood (2); Sputum (1); Liquor puris (1)
LOD^d^	2 (2.17)	Male (1), Female (1)	15, 30 d	Blood (2)
Bronchopneumonia	2 (2.17)	Female	5	Sputum (2)
Inevitable abortion	1 (1.09)	Female	33	Cervical secretions (1)
Premature delivery	1 (1.09)	Female	28	Cervical secretions (1)
Late production	1 (1.09)	Female	33	Cervical secretions (1)
Ectopic pregnancy	1 (1.09)	Female	31	Cervical secretions (1)
Infection of cesarean section	1 (1.09)	Female	34	Liquor puris (1)
Chronic suppurative sinusitis	1 (1.09)	Female	12	Liquor puris (1)
Diabetes mellitus	1 (1.09)	Female	54	Urine (1)
Cardiovascular disease	1 (1.09)	Female	83	Vaginal secretions (1)
Male infertility	1 (1.09)	Male	36	Urine (1)
Dermatosis	1 (1.09)	Male	17	Liquor puris (1)
Fever	1 (1.09)	Male	82	Blood (1)

a *GBS, group B Streptococcus*.

b *All pregnant women are full-term pregnancy*.

c *EOD, early-onset disease*.

d *LOD, late-onset disease*.

### Molecular serotyping

Molecular serotyping was performed as previously reported (Poyart et al., 2007; Li et al., 2013). PCR products were purified and sequenced (TaKaRa, China). The sequencing results were analyzed in NCBI (http://www.ncbi.nlm.nih.gov).

### Pulsed-field gel electrophoresis (PFGE)

PFGE assay was performed as previously reported (Chen et al., 2012). PFGE patterns were analyzed and compared using the BioNumerics (Applied Maths, Belgium).

### Multilocus sequence typing (MLST)

Seven housekeeping genes (adhP, pheS, atr, glnA, sdhA, glcK, and tkt) were PCR amplified and sequenced as described previously (Jones et al., 2003). The allele number, sequence types (STs) and CC analysis were performed using MLST database (http://pubmlst.org/sagalactiae/) and eBURST program (http://eburst.mlst.net). The allele sequences and ST previously unreported were assigned with new numbers in the MLST database.

### Antimicrobial susceptibility

All the 92 isolates were tested for susceptibility to penicillin, erythromycin, clindamycin, vancomycin, and tetracycline (Oxoid) using disk diffusion method according to the Clinical and Laboratory Standard Institute (CLSI) guidelines (CLSI, 2010).

### Pilus island genes and gbs2018 genes

Virulence factors PI-1, PI-2a, and PI-2b were detected as previously reported (Madzivhandila et al., 2013). Cluster typing of highly virulent gene gbs2018 was performed as previously reported (Lamy et al., 2006; Tazi et al., 2010). Table S2 listed all the primers used in the test.

### Statistical analysis of CC103 strains in literature and MLST database

**Figure 1:**
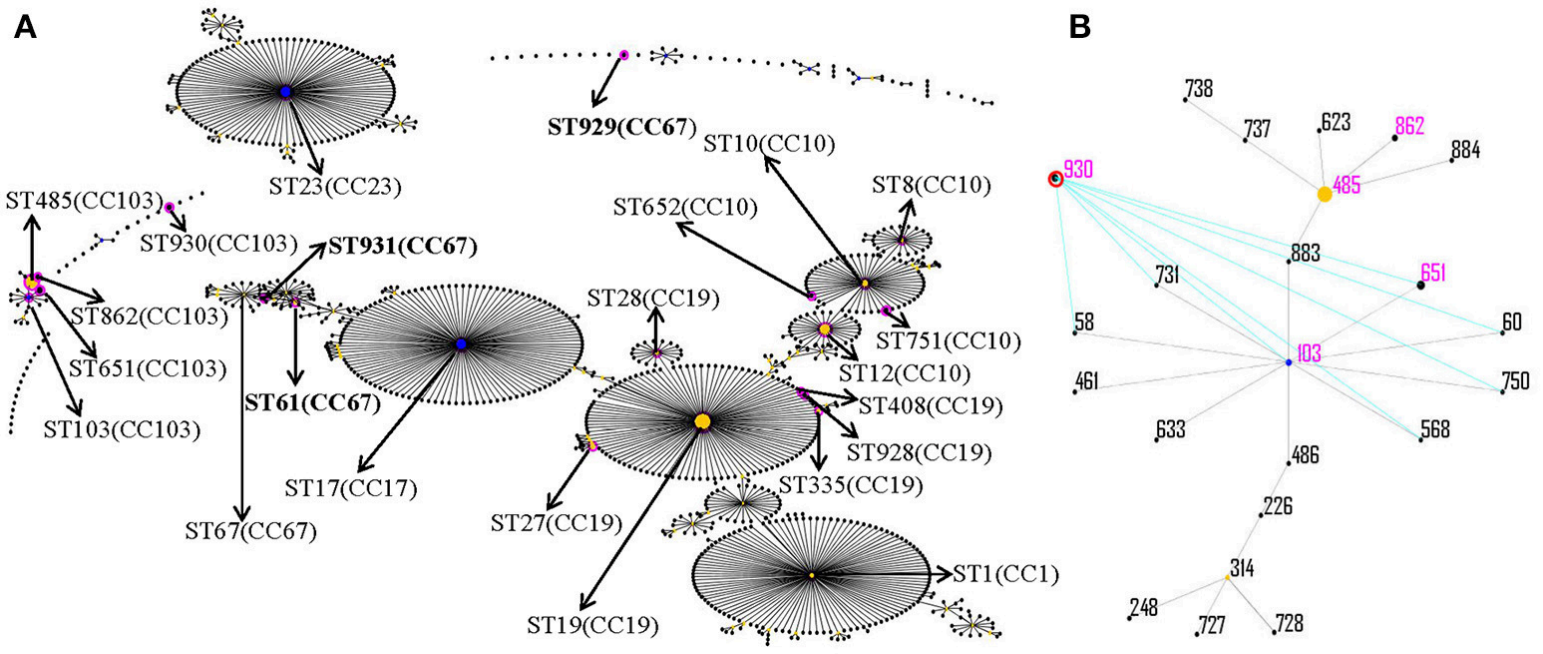
eBURST analysis of *S. agalactiae* using all STs available in the MLST database as of January 2017. Each sequence type (ST) is represented as a dot. The dots positioned centrally in the cluster are primary founders (blue) or subgroup founders (yellow), and the area of each circle indicates the prevalence of the ST in this study. Pink circles indicate STs detected from the strains in this study and are marked by solid line arrows. For clarity, ST labels have been removed. **(A)** eBURST analysis of *S. agalactiae* using all STs available in the MLST database as of January 2016; **(B)** all ST types of CC103.

To investigate the detection rate of CC103 and ST485 in public literature, we searched PubMed database from January 1, 2000 to October 1, 2016 using keywords *Streptococcus agalactiae*, Streptococcus Group B, Group B Streptococcus, in combination with Multilocus sequence typing, Multilocus sequence type, and MLST, and extracted information from literatures meeting the following criteria: (1) the study subject is *S. agalactiae* isolated from human, bovine, fish, and other animals or environment specimens; (2) performed MLST typing and detected one or more STs of CC103, and (3) ST detection rate could be calculated. In addition, only the latest and most comprehensive published data were selected for repeated literature of the same author or agency. The information of the included literature was listed in Table 1. Moreover, we also searched each ST (Figure 1) of CC103 in the isolates database of *S. agalactiae* MLST database and extracted the information of every retrieved strain (Table 1).

### Evolutionary relationship analysis of CC103 strains based on whole genome

The draft genome sequences of *S. agalactiae* strains C001, BSE009, LZF004, NNA006, BSE003, and BSE004 were determined using Illumina HiSeq2000 at Novogene Bioinformatics Institute (Novogene, China) and assembled using the ABySS 1.9.0 program. The accession numbers of C001, BSE009, LZF004, NNA006, BSE003, and BSE004 are listed in Table S3. In addition, 39 major strains of *S. agalactiae* from human, bovine and fish and three other animal-derived strains in the public database were selected for evolutionary analysis (Table S3). OrthoMCL was used to delineate orthologous protein sequences among the isolates. Multiple sequence alignment of single copy homologous protein sequence was performed using MAFFT to remove the poorly aligned positions and divergent regions. The optimal amino acid substitution model was obtained by comparing the AIC and BIC scores. The maximum likelihood method was used to construct a phylogenetic tree with 1,000 bootstrap replications using RaxML software.

### Experimental infection

Non-infected Nile tilapia (Oreochromis niloticus, 90–120 days age) with an average weight of 50.96 ± 9.38 g were provided by the National Tilapia Seed Farm (Nanning, China). Infection assay was performed strictly according to our previous protocol (Chen et al., 2015). The 92 GBS isolates and one tilapia GBS strain HN016 were used to infect tilapia at 1.0 × 10^9^ and 1.0 × 10^6^ CFU/fish. A total of 10 fish in each group were intraperitoneally (IP) injected with 0.1 mL of every strain. The blank control group was injected with 0.1 mL of sterile PBS. Challenged fish were monitored and fed twice a day for 15 days. The bacteria were re-isolated and identified as described above from brain and liver samples collected from all dead and survival fish at the end of the experiment.

### Tissue pathology

At 24 h of post-infection, the infected tilapia were euthanized sand their brain, liver, spleen, head-kidney, and posterior intestine were collected and placed into 10% neutral buffered formalin. After fixation, the organs were embedded in paraffin, sliced as 5 μm sections, and stained with hematoxylin and eosin for histological evaluation.

### Statistical analysis

SPSS software (version 17.0) was used for data analysis. Student *t*-test and the Wilcoxon rank-sum test were used for analysis of continuous variables. Continuous variables were compared using the Spearman ρ correlation analysis. Categorical variables were compared using the χ2 test or Fisher exact test.

## Results

### Invasive GBS isolates

Ninety-two isolates from 92 patients with 19 different diseases (Table 1) were identified as *S. agalactiae*. Among the 92 patients, vaginitis (22.83%), threatened abortion (16.30%), premature rupture of membranes (15.22%), cervicitis (9.78%), urethritis (8.70%), and pelvic inflammation (6.52%) are the most frequent diseases of pregnant/puerperal women, accounting for 79.35%. Based on the statistics of culture source, cervical secretions (30.87%), vaginal secretions (23.91%), amniotic fluid (16.30), and urine (6.52%) were the main GBS source of pregnant/puerperal women, accounting for 82.61%.

### Distribution of serotypes and STs

The results of serotypes and STs are summarized in Table 2. Of the 92 collected GBS isolates, the most predominant serotypes were serotypes III, Ia, V and Ib, accounting for 34.78% (32/92), 28.26% (26/92), 16.30% (15/92), and 14.13% (13/92), respectively, followed by serotypes II (6, 6.52%). The serotypes IV, VI, VII, and VIII were not found. eBURST V3 analysis (Figure 1) revealed that the 92 GBS isolates were resolved into 7 clonal complexes (CCs) with 22 different STs. The predominant STs of the isolates were identified as ST19 (23, 25.00%), ST485 (13, 14.13%), ST23 (10, 10.87%), ST17 (10, 10.87%), and ST12 (10, 10.87%). Additionally, four new STs (ST928, ST929, ST930, and ST931) and three new allele sequences (atr-110, glnA-96, sdhA-93) were identified.

**Table 2:** Distribution of serotypes and STs of 92 invasive GBS strains from different disease patients.

**CC (*n*, % ^a^)**	**ST (*n*, %^a^)**	**No. of isolates with serotype**											**No. of isolates with disease of patient**													
		**Ia**	**Ib**	**II**	**III**	**V**	**A**	**B**	**C**	**D**	**E**	**F**	**G**	**H**	**I**	**J**	**K**	**L**	**M**	**N**	**O**	**P**	**Q**	**R**	**S**	**T**
1 (2, 2.17)	1 (2, 2.17)					2			2																	
12 (18, 19.57)	8 (1, 1.09)		1						1																	
	10 (4, 4.35)		2	2			2								2											
	12 (10, 0.87)^*^		10^*^				2	2			1						1	1	1			1				1
	652 (1, 1.09)			1				1																		
	751 (2, 2.17)			2			1					1														
17 (10, 10.87)	17 (10, 10.87)				10^*^				2	1		1	3^*^	2										1		
19 (29, 31.52)	19 (23, 25.00)^*^				12^*^	11^*^	4^*^	2	6^*^	3^*^	2	3^*^				1							1		1	
	27 (2, 2.17)				2			1			1															
	28 (1, 1.09)			1						1																
	335 (1, 1.09)				1															1						
	408 (1, 1.09)				1			1																		
	928 (1, 1.09)				1			1																		
23 (10, 10.87)	23 (10, 0.87)^*^	10					4^*^		1	1	3^*^	1														
	61 (1, 1.09)				1		1																			
67 (3, 3.26)	929 (1, 1.09)					1		1																		
	931 (1, 1.09)					1					1															
103 (20, 21.74)	103 (1, 1.09)	1								1																
	485 (13, 14.13)^*^	13					4^*^	4^*^	2	1			1								1					
	651 (3, 3.26)				3		1	1		1																
	862 (1, 1.09)				1			1																		
	930 (2, 2.17)	2					2																			
Total	92	26	13	6	32	15	21	15	14	9	8	6	4	2	2	1	1	1	1	1	1	1	1	1	1	1

*A–T, Representing 19 diseases; A, Vaginitis; B, Threatened abortion; C, Premature rupture of membranes; D, Cervicitis; E, Urethritis; F, Pelvic inflammatory disease; G, EOD; H, LOD; I, Bronchopneumonia; J, Inevitable abortion; K, Premature delivery; L, Late production; M:Ectopic pregnancy; N, Infection of cesarean section; O, Chronic suppurative sinusitis; P, Diabetes mellitus; Q, Cardiovascular disease; R, Male infertility; S, Dermatosis; T, Fever*.

a *n, No. of isolates*.

* *Indicates that no. of isolates with ST or diseases are significantly larger than their mean values*.

### Pulsed-field gel electrophoresis (PFGE) analysis

**Figure 2:**
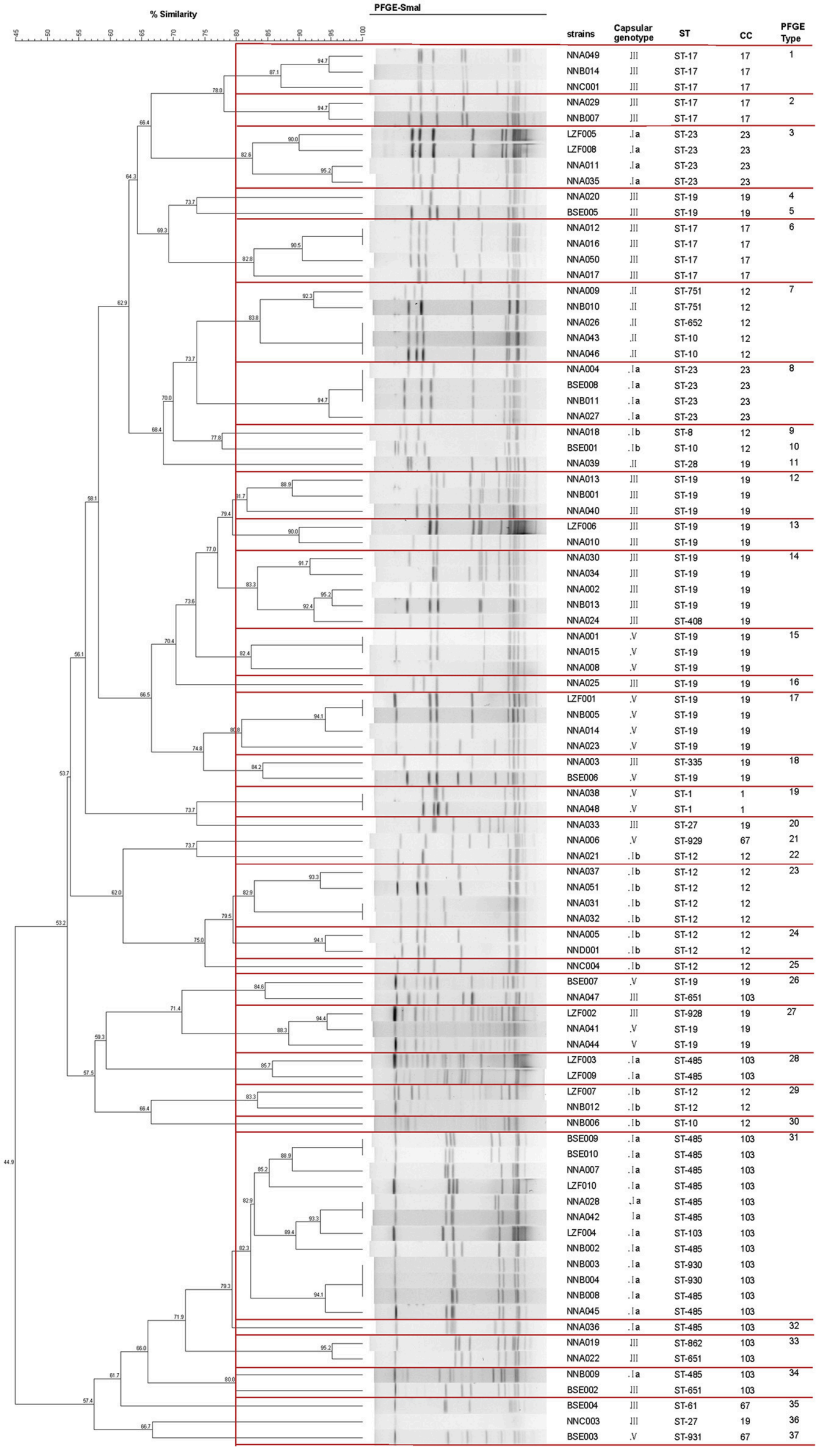
Dendrogram of PFGE profiles of 92 invasive *S. agalactiae*. The Dendrogram was constructed based on BioNumerics analysis of the *S. agalactiae* PFGE patterns and cluster analysis using the Dice coefficient and UPGMA of the digitalized PFGE patterns for the *S. agalactiae* strains. Clustering settings of 0.00% optimization and 1.5% band position tolerance were used. Dice coefficients (percentages) are indicated in the scale above the dendrogram. Each cluster (defined as a group of two or more isolates with a Dice coefficient of ≥80%) is represented in the dendrogram.

The isolates were grouped in 37 PFGE clusters according to 80% similarity on the dendrogram (Figure 2). Most isolates of CC103 (85.00%, 17/20) and CC19 (68.97%, 20/29) were clustered in the same branch with a similarity >66%. By contrast, the PFGE clustering of CC12\17\23 strains was relatively dispersing. The concordance between PFGE-based genotypes and the eBURST-based genotypes showed that 100% of all pairs of isolates in the same PFGE also share the same CCs. The concordance between PFGE-based genotypes and the serotypes showed that 94.57% (87/92) of any pair of isolates in the same PFGE also share the same serotype.

### Prevalence of CC103 and ST485 isolates from human is significantly higher than that reported in the literature

Statistical analysis showed that CC103 strains were not identified in 76.47% (65/85) of literature and CC103 strains were detected in 20 reports (Table 3). Among the 20 reports, the prevalence was 0.1-2.3% for human CC103 strains (except 4.6–7.1% in two reports from China), 0.6–7.1% for human ST485, which were only detected in three reports, 0.5–88.3% for bovine CC103 and 0 for bovine ST485. Among the 92 invasive GBS (Table 3), the prevalence was 21.74% (20/92) for CC103 strain and 14.13% (13/92) for ST485. The prevalence of CC103 and ST485 in this study was two to three times higher than that in Beijing/Shanghai and 10-20 times higher than that in other area out of China. Among the 146 CC103 strains in the literature (Table 1), 68.49% (100/146) were pathogenic strains, 4.11% (6/146) were carrier strains and 27.4% (40/146) were undetermined.

**Table 3:** The information of CC103 isolates obtained through searching literature and MLST database.

**Route**	**Ref./ isolate/ID^a^**	**ST**	**Year**	**No. /%^b^**	**Country/area**	**Host/Culture source**	**Invasive/carrier**
Literature	Jiang et al., 2016	103	2016	2/2.3	China/Shanghai	Human/unknown	Unknown
		485		2/2.3		Human/unknown	Unknown
	Parker et al., 2016	103	2016	1/0.3	USA	Humans or cattle/unknown	Unknown
	Jorgensen et al., 2016	103	2016	16/28.6	Norway	Bovine/milk	Unknown
		731		4/7.1		Bovine/milk	Unknown
	Wang et al., 2015	485	2015	4/7.1	China/Beijing	Human/rectal and vaginal	Unknown
	Lu et al., 2015	485	2015	2/1.25	China/Beijing	Human/rectal and vaginal	Unknown
		486		1/0.6		Human/rectal and vaginal	Unknown
	Usein et al., 2014	103	2014	1/1.8	Romania /Bucharest	Human/vaginal	Carrier
	Godoy et al., 2013	103	2013	2/4.34	Brazil	Fish/unknown	Invasive
	Yang et al., 2013	103	2013	58/56.9	Eastern China	Bovine/milk	Invasive
		568		32/31.4		Bovine/milk	Invasive
	De Francesco et al., 2012	461	2012	1/1.8	Italy/Brescia	Human/vaginal and urine	Unknown
	Huber et al., 2011	103	2011	1/0.6	Kenya/Nairobi	Human/unknown	Invasive
		485		1/0.6		Human/unknown	Invasive
		486		2/1.2		Human/unknown	Invasive (1) /carrier (1)
	Haguenoer et al., 2011	314	2011	1/0.5	France	Humans or cattle/unknown	Unknown
	Springman et al., 2009	103	2009	1/1.1	Canada	Human/unknown	Carrier
	Lartigue et al., 2009	226	2009	1/0.5		Human/unknown	Unknown
		314		1/0.5		Human/unknown	Unknown
	Eickel et al., 2009	103	2009	1/1.1	Germany/Münster	Human/respiratory	Invasive
		314		1/1.1		Human/respiratory	Invasive
	Manning et al., 2009	103	2009	1/0.5	Canada/Alberta	Human/unknown	Invasive
	Bohnsack et al., 2008	103	2008	1/0.1	USA/ SanoPaulo	Human/unknown	Carrier
	Oliveira et al., 2006	103	2006	1/5.0	USA	Bovine/milk	Invasive
	Marchaim et al., 2006	103	2006	1/1.4	Southern Israel	Human/unknown	Carrier
	Brochet et al., 2006	103	2006	3/4.0	–	Pig/nose, Cow, cat/urine	Unknown
		248		1/1.3		Human/urine	Unknown
	Jones et al., 2006	103	2006	2 /0.5	United Kingdom	Human/unknown	Carrier (1), Invasive (1)
MLST database	BSU10/1177	226	2015	1	Unknown	Unknown	Unknown
	GBS222/1772	862	2015	1	Singapore	Human/blood	Invasive
	C67/1785	884	2015	1	China	Human/vaginal	Carrier
	H003/961	737	2015	1	China	Human/vaginal	Carrier
	H010/962	738	2015	1	China	Human/vaginal	Carrier
	BSU451/1192	103	2015	1	Unknown	Unknown	Unknown
	MRI Z1-023/1375	103	2015	1	Unknown	Unknown	Unknown
	NNB003/1825	930	2015	1	China	Humans/unknown	Invasive
	6_3/971	731	2015	1	Norway /Oppland	Bovine/milk	Invasive
	6_3/955	731	2013	1	Norway /Oppland	Bovine/milk	Invasive
	MRI Z2-137/ 1462	750	2012	1	Finland	Human/urinary	Invasive
	S286/874	651	2012	1	China	Human/unknown	Unknown
	MRI Z2-039/855	633	2012	1	Finland	Cows/milk	Invasive
	ky65/846	623	2012	1	China	Human/rectovaginal	Carrier
	MRI Z2-157/951	727	2012	1	Sweden	Bovine/milk	Invasive
	MRI Z2-174/952	728	2012	1	Sweden	Bovine/milk	Invasive
	A11/342	568	2011	1	China/jiangsu	Bovine/milk	Invasive
	694/694	103	2011	1	Thailand	Bovine/milk	Invasive
	697-705/697705	103	2011	9	Thailand	Bovine/milk	Invasive
	C8/1784	883	2009	1	China	Human/vaginal	Carrier
	10-09_cDK/48	461	2009	1	Denmark	Bovine/milk	Invasive

a *Ref, Reference*.

b *No. of isolates with ST and its percentage in total isolates researched in the literature*.

### Antimicrobial resistance

The results of antimicrobial susceptibility test showed that all 92 strains of GBS were sensitive to penicillin and vancomycin, 60.87% (56/92) strains were resistant to erythromycin and 52.17% (48/92) were resistant to clindamycin. Among them, 42 (45.65%) were resistant to both erythromycin and clindamycin and as high as 96.74% (89/92) strains were resistant to tetracycline. Strain drug resistance has a certain correlation with serotype and CC group (Table 4).

**Table 4:** Distribution of Antimicrobial resistance among 92 GBS according to CC, MS.

**CC MS**\**Antibiotic**		**Erythromycin**	**Clindamycin**	**Tetracycline**
CC103	AR, No.	11/20 (55%)	6/20 (30%)	17/20 (85%)
	OR, No.	44/72 (61%)	42/72 (58%)	72/72 (100%)
	*P-*value	0.404	0.023^*^	0.009^*^
CC19	AR, No.	20/29 (69%)	16/29 (55%)	29/29 (100%)
	OR, No.	35/63 (56%)	32/63 (51%)	60/63 (95%)
	*P-*value	0.161	0.435	0.316
CC23	AR, No.	0/10 (0%)	2/10 (20%)	10/10 (100%)
	OR, No.	55/82 (67%)	46/82 (56%)	79/82 (96%)
	*P-*value	<0.001^*^	0.033^*^	0.705
CC17	AR, No.	10/10 (100%)	10/10 (100%)	10/10 (100%)
	OR, No.	45/82 (55%)	38/82 (46%)	79/82 (96%)
	*P-*value	0.004^*^	0.001^*^	0.705
CC12	AR, No.	11/18 (61%)	12/18 (67%)	18/18 (100%)
	OR, No.	44/74 (59%)	36/74 (49%)	71/74 (96%)
	*P-*value	0.56	0.133	0.516
CC1	AR, No.	2/2 (100%)	2/2 (100%)	2/2 (100%)
	OR, No.	53/90 (59%)	46/90 (51%)	87/90 (97%)
	*P-*value	0.355	0.269	0.935
CC67	AR, No.	1/3 (33%)	0/3 (0%)	3/3 (100%)
	OR, No.	54/89 (61%)	48/89 (54%)	86/89 (97%)
	*P-*value	0.354	0.105	0.904
Ia	AR, No.	10/26 (39%)	6/26 (23%)	24/26 (92%)
	OR, No.	46/66 (70%)	42/66 (64%)	65/66 (99%)
	*P-*value	0.006^*^	<0.001^*^	0.192
Ib	AR, No.	11/13 (85%)	12/13 (92%)	13/13 (100%)
	OR, No.	45/79 (57%)	36/79 (46%)	76/79 (96%)
	*P-*value	0.052	0.001^*^	0.63
II	AR, No.	1/6 (17%)	0/6 (0%)	6/6 (100%)
	OR, No.	55/86 (64%)	48/86 (56%)	83/86 (97%)
	*P-*value	0.032^*^	0.01^*^	0.815
III	AR, No.	21/32 (66%)	23/32 (72%)	31/32 (97%)
	OR, No.	35/60 (58%)	25/60 (42%)	58/60 (97%)
	*P-*value	0.325	0.005^*^	0.724
V	AR, No.	13/15 (87%)	7/15 (47%)	15/15 (100%)
	OR, No.	43/77 (56%)	41/77 (53%)	74/77 (96%)
	*P-*value	0.022^*^	0.426	0.582

*CC, Clonal complexes; MS, molecular serotype; AR, antibiotic resistance of isolates; OR, all other isolates*.

* *P < 0.05 compared with OR*.

### Distributional characteristics of virulence-associated factors

#### Pilus islands

**Figure 3:**
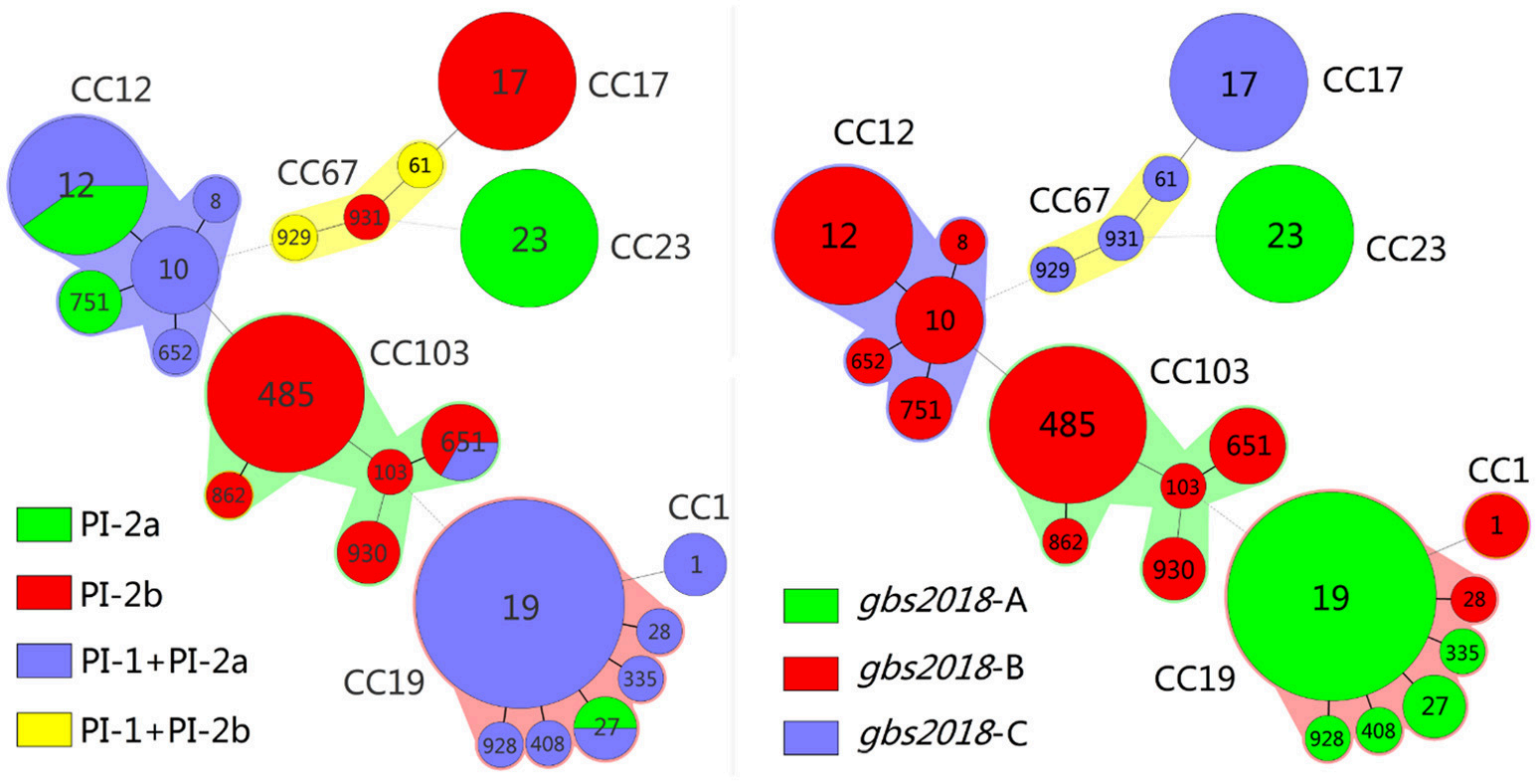
Correlation between clonal complexes (CCs) and sequence type (ST) with pilus island (PI) and Gbs2018 gene of 92 GBS isolates in this study. Minimum spanning tree analysis of GBS isolates according to ST, demonstrating the relationships between 92 invasive isolates collected from three cities of China. In the minimum spanning tree, the STs are displayed as circles, and the area of each circle indicates the prevalence of the ST in this study. The founder ST was defined as the ST with the greatest number of single-locus variants. Pilus islands profiling and Gbs2018 genes are represented by different colors in the left and right figures, respectively. STs that vary by one or two allele in their multilocus sequence typing (MLST) profiles (single-locus variants) are arranged in circles around the primary founder ST. Heavy solid lines represent singlelocus variants; light solid lines represent double-locus variants; heavy dotted lines represent triple-locus variants; light dotted lines represent quadruple-locus variants. The major CCs are indicated in the figure.

The pilus islands of *S. agalactiae* have four types: PI-2a, PI-2b, PI-1+PI-2a, and PI-1+PI-2b. PCR results showed that the 92 isolates belong to at least one of the 4 types (Figure 3). PI-2a was the most common type, followed in turn by PI-1+PI-2a, PI-2b, and PI-1+PI-2b. The type of pilus islands was strongly correlated with ST and serotype. All ST17 (10/10, 100%) and ST485 (13/13, 100%) were PI-2b, all ST23 (10/10, 100%) were PI-2a, and all ST19 (23/23, 100%) and ST10 (4/4, 100%) were PI-1+PI-2a. Only two isolates, both of which were CC67, were PI-1+PI-2b. In addition, the correlation analysis of diseases and types of pilus islands found (Table S5) that the correlation of PI-2a to threatened abortion and urethritis was strong (*P* = 0.035 and *P* = 0.005). Meanwhile, the correlation of PI-2b to EOD and LOD was also strong (*P* = 0.01 and *P* = 0.048).

#### gbs2018 gene

PCR analysis (Figure 3) showed 38 strains carrying gbs2018-A gene, 41 strains carrying gbs2018-B gene and 13 strains carrying gbs2018-C gene. gbs2018 gene type is highly correlated with CC group. All CC23 (10/10, 100%) and most CC19 (28/29, 96.6%) carried gbs2018-A; all CC12 (18/18, 100%), CC103 (20/20,100%), and CC1 (2/2, 100%) carried gbs2018-B and all CC17 (10/10, 100%) and CC67 (3/3, 100%) carried gbs2018-C. In addition, the correlation analysis of diseases with types of gbs2018 gene found (Table S6) that the correlations of gbs2018-A to urethritis (*P* = 0.05), and gbs2018-C to EOD (*P* = 0.008) and LOD (*P* = 0.019) were strong.

### Genetic evolutionary relationships of CC103 and CC67 strains

**Figure 4:**
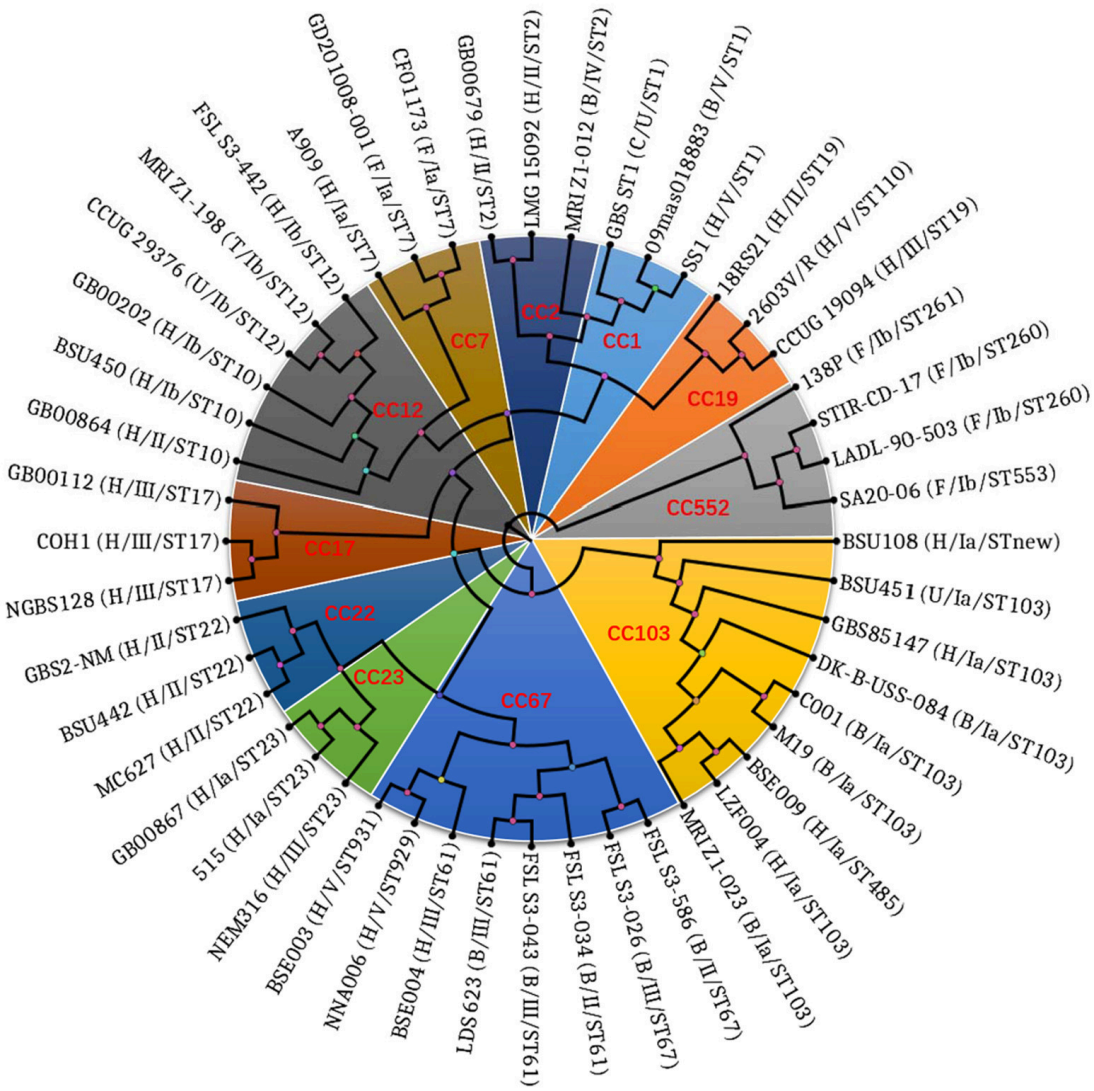
Phylogenetic tree showing the relationship of 48 *S. agalactiae* strains based on the 159,419 amino residual positions of 789 single copy ortholog groups. The Maximum Likelihood (ML) method was used to construct the phylogenetic tree. The tip labels are strain number, host, and serotype. Letters H, B, F, C, T, and U in the brackets of tip labels are abbreviations of Homo sapiens, Bos Taurus, Fish, Canis lupus familiaris, Tursiops, and unknown, respectively. Those marked in red are the clonal complexes.

The phylogenetic tree showed that strains with the same CC group had close evolutionary relationship (Figure 4). It is interesting to note that none of the CC groups exhibited host specificity unless they were from the same host. Strains with the same CC groups but from different hosts were also grouped into one evolutionary branch, such as CC7 from human and fish. It is noteworthy that the three isolates NNA006, BSE003 and BSE004 from human were grouped into one evolutionary branch with CC67, which had been considered specific to cows. In the evolutionary branch, CC103 constituted an independent evolutionary branch, while bovine and human strains did not form a host independent evolutionary branch and human and bovine ST103 strains underwent staggered evolution.

### Tilapia infection test

**Figure 5:**
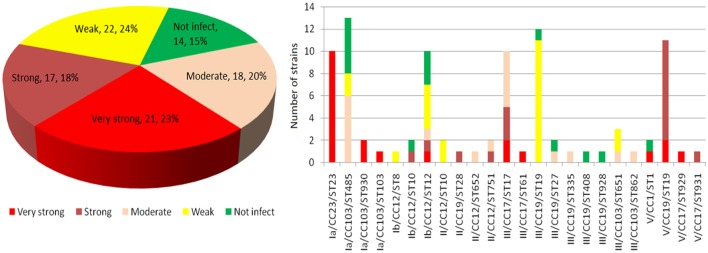
Results of Tilapia infection test with 92 human invasive GBS. 92 human invasive GBS isolates were used for intraperitoneally injecting 10 tilapia per strain per high dose of 1.0 × 10^9^ CFU/fish and low dose of 1.0 × 10^6^ CFU/fish. The figure shows the results of injecting GBS strains at high dosage. No, weak, moderate, strong and very strong indicate zero, 1–3, 4–6, 7–8, and 9–10 tilapia died during the experiment. The number of died tilapia per strain is shown in Table S1.

According to the mortality rate at high (1.0 × 10^9^ CFU/fish) dose (Figure 5 and Table S4), pathogenicity of 92 human GBS isolates to tilapia can be divided into five categories, namely very strong, strong, moderate, weak, and no cross host infection ability, which had mortality rate of 90–100% (21 isolates), 70–80% (17), 40–60% (18), 10–30% (22), and 0% (14), respectively. At low (1.0 × 10^6^ CFU/fish) dose, 22 isolates with weak cross host infection ability and 14 isolates with no cross host infection ability resulted in death of zero out of 10 tilapia. Further analysis (Figure 5) revealed that the pathogenicity of 92 human GBS to tilapia was greatly associated with their ST/CC serotypes. All 10 ST23 isolates had very strong pathogenicity to tilapia. Seventeen CC19 isolates with serotype III had no or weak pathogenicity to tilapia. By contrast, 11 CC19 isolates with serotype V and one CC19 isolate with serotype II had very strong or strong pathogenicity to tilapia. In addition, different ST type of CC103 isolates had very different pathogenicity to tilapia. ST103 isolates had very strong pathogenicity to tilapia, while ST485 isolates had no or weak pathogenicity to tilapia. All tilapia died due to infection had clinical signs of lethargy, anorexia, erratic swimming, exophthalmia, and bleeding at fin base. Moreover, a large number of single GBS strain could be isolated from the brain and liver of the dead tilapia, while no GBS strain and other bacteria were isolated from the brain and liver of the survival tilapia.

### Pathological analysis

**Figure 6:**
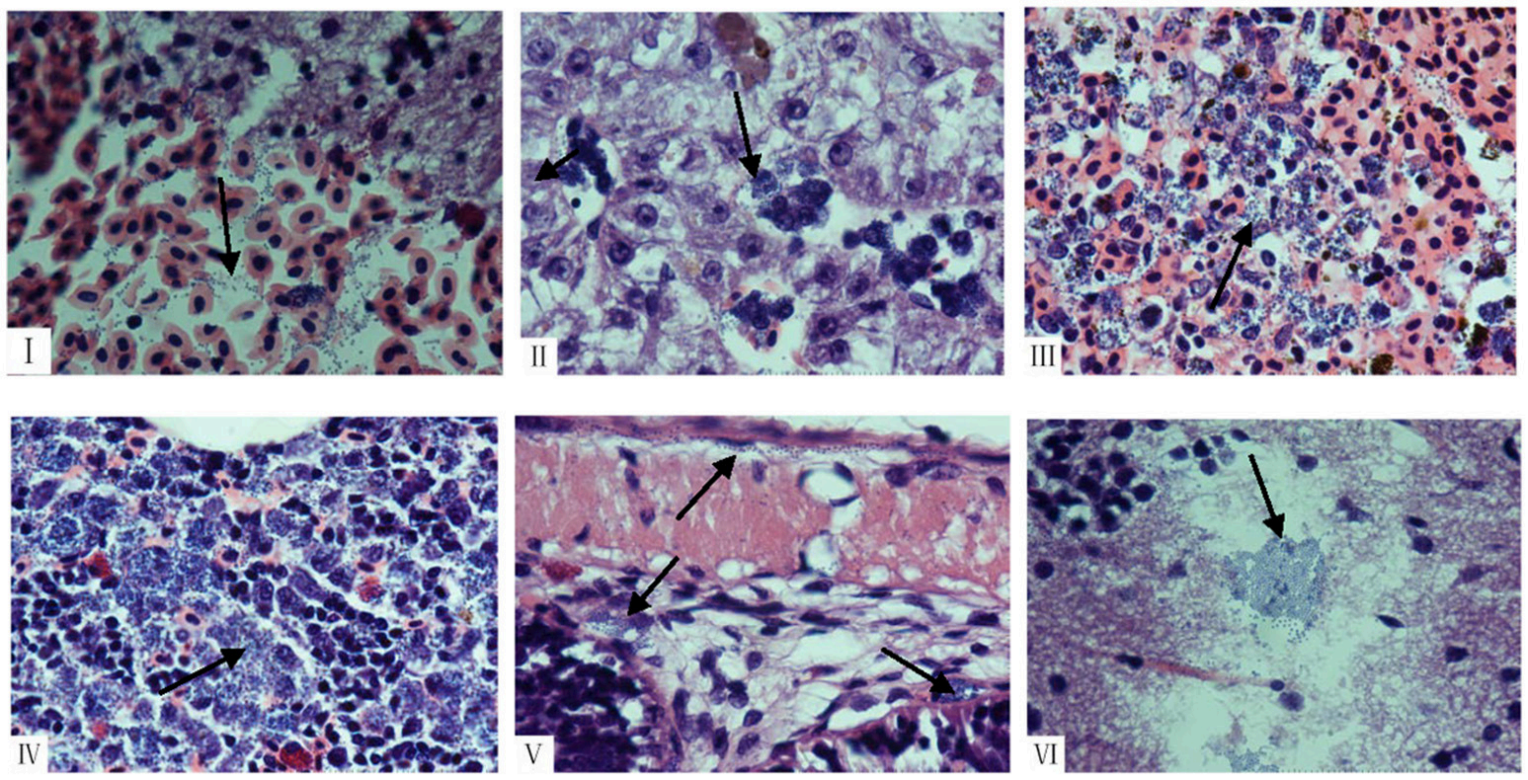
Histopathological changes shown by HandE staining in collected organs from tilapia infected with NNA048 and HN016 at 24 h of post infection. HE images at 1,000 × magnification of brain, liver, spleen, head-kidney, and intestine of infected tilapia with human NNA048 and tilapia HN016 at 24 h of post infection. **(I)** The brains of NNA048 infected tilapia, showing brain hemorrhage and blue-stained streptococcus particles; **(II)** The liver of NNA048 infected tilapia, showing hemosiderin deposition and necrosis lesions with a large amount of blue-stained streptococcus particles inside; **(III)** The spleen of NNA048 infected tilapia, showing bleeding, necrosis lesions with a large amount of blue-stained streptococcus particles inside, and scattered hemosiderin deposition; **(IV)** The head-kidney of NNA048 infected tilapia, showing blurry structure, significantly reduced lymphocytes, as well as a large amount of necrotic lesions with a large number of blue-stained streptococcus particles; **(V)** The intestine of NNA048 infected tilapia, showing blurry serous layer boundary, and blue-stained streptococcus particles in serosa, myometrium as well as submucosal and mucosal layers; **(VI)** The brains of NNA016 infected tilapia, showing a large amount of blue-stained streptococcus particles.

Pathological analysis showed that there was no significant difference between tilapia infected with human NNA048 and tilapia HN016 except the number of pathogens was more in the tissues infected with tilapia HN016 than that infected with human NNA048 (Figure 6). NNA048 could break through the blood-brain barrier into tilapia brain tissue and cause pathological changes in major organ tissues as that of HN016.

## Discussion

GBS are not only common invasive strains in human newborns and female urinary system, but also common colonizing strains of human digestive tract, respiratory tract and urogenital tract (Bohnsack et al., 2008; Lartigue et al., 2009; Manning et al., 2009). Our results (Table 1) showed GBS could be isolated from pregnant/puerperal women patients with 15 kinds of diseases and the top three were vaginitis (22.83%), threatened abortion (16.30%) and premature rupture of membranes (15.22%). Meanwhile, our results also showed that each disease had its own major serotype/ST-type strains and ST12 could be found in most diseases (Table 2). It is worth noting that the prevalence of CC103 strains (21.74%) and ST485 (14.13%) in our study is significantly higher than those reported previously. Furthermore, CC103/ST485 have become the dominant ST in the top three diseases of pregnant/puerperal patients and also discovered in EOD of newborns. In addition, almost all CC103, CC17 and CC67 strains in pilus islands were PI-2b or PI-1+PI-2b type (Figure 3), and the ratio of PI-2b was significantly higher than that in other reports in China (Lu et al., 2015, 2016). These results may be related to the pathogenic strain used in this study. Therefore, the phenomena of significantly increased prevalence and pathogenicity of CC103 and ST485 strains should be highly concerned.

In order to analyze the prevalence of CC103/ST485 in human, cows and other animals, we statistically analyzed literature and MLST database (Table 3) and found the prevalence of CC103 and ST485 in this study was two to three times higher than that in Beijing/Shanghai and 10–20 times higher than that in other area out of China (Zadoks et al., 2011; Yang et al., 2013; Wang et al., 2015; Jiang et al., 2016). The reason for such high prevalence of CC103 and ST485 in the current study was most likely that invasive strains were collected in the current study while the clonal strains were used in literature. In addition, the results also showed that 1) ST485 strain and its all SLV (ST883\ST884\862\623\737) strains were human-derived and most ST485 strains were pathogenic; 2) ST103 and its SLV, DLV (except ST485), and TLV stains were all detected in human and dairy cows; and 3) ST103 and ST568 strains were the absolute dominant strains in dairy cows and most of them are pathogenic. Meanwhile, other studies found that ST103 strain which was barely detected in dairy cows before has become the dominant ST in dairy cows in Europe and some countries in East Asia (Zadoks et al., 2011; Yang et al., 2013), and ST103 strain was also isolated from pigs, cats, fish and other animals in the last decade (Brochet et al., 2006; Godoy et al., 2013; Zhang et al., 2017). Further evolutionary analysis based on the WGSs showed that all CC103 including ST485 strains constituted independent evolutionary branches, and the bovine and human ST103 strains formed stellate rather than independent host evolutionary branches (Figure 4). All above indicated that CC103 strain is able to infect human, dairy cows, pig, cat, fish, and other wide-spectrum hosts, and has developed to highly pathogenic human ST485 strain and dairy cow ST103 and ST568 strains. Although the prevalence of pathogenic ST485 is only significantly increased in Guangxi, Beijing and Shanghai, not in other regions, the prevalence of dairy cows ST103 has become globalized (Da Cunha et al., 2014; Wang et al., 2015; Jiang et al., 2016; Parker et al., 2016).

Whether the hyper-invasive human neonatal clone ST17 was originated from dairy cow CC67 has been controversial (Tongs, 1919; Meleney, 1927; Bisharat et al., 2004; Sørensen et al., 2010; Zadoks et al., 2011). The results of eBURST analysis showed that ST17 was arisen from bovine ST67 (Bisharat et al., 2004). But most scholars believed that human and bovine *S. agalactiae* are largely distinct populations (Dogan et al., 2005; Duarte et al., 2005; Sukhnanand et al., 2005; Brochet et al., 2006; Sørensen et al., 2010). In this study, evolutionary analysis based on MLST and WGSs confirmed that the three isolates NNA006 (ST929), BSE003 (ST931), and BSE004 (ST61) were originated from dairy cow CC67 strain. However, the three isolates have undergone major variations. Among them, ST931 is the DLV of ST61 and ST929 is the DLV of ST931. Otherwise, the serotype of dairy cow CC67 strain is Ia or II, while that of NNA006 and BSE003 is V and BSE004 is III. However, the genotype of the three isolates in pilus islands is consistent to that of the dairy cow CC67 strain, which was PI-2b (Figure 3). It is particularly noteworthy that the three isolates have acquired gbs2018-C gene, which is specific to the highly pathogenic CC17 strain (Figure 3) and also known as hypervirulent GBS adhesin (HvgA) gene. The gene is directly linked to the high pathogenicity of CC17 strain (Lamy et al., 2006; Tazi et al., 2010). These characteristics of the three isolates make it necessary to rethink the origin of CC17 strain. The three isolates were probably in the transition state of CC67 strain to CC17 strain. Further analysis of the evolutionary relationship between the three isolates and CC67/CC17 supported this assumption (data unpublished). This is the first time isolation of human CC67 isolate that carries CC17 strain-specific HvgA gene. Therefore, the CC67 GBS of dairy cows has a huge threat to public health and safety of people.

Although fish *S. agalactiae* is prevalent in the world, the ST or CC types of fish GBS strains were relatively stable and ST7 and ST260/261 were the predominant ST (Godoy et al., 2013; Li et al., 2013; Liu et al., 2013; Rosinski-Chupin et al., 2013). In recent years, CC283 and ST103 strains were isolated in Thailand, China and Brazil (Delannoy et al., 2013; Godoy et al., 2013; Zhang et al., 2017). Since the genome sequences of CC7 strains and a small number of ST103 and ST283 strains are highly homologous to that of human-derived ST-type strains, it is inferred that these three ST strains were derived from human (Liu et al., 2013; Tan et al., 2016). This study systematically analyzed the pathogenicity of 92 human invasive GBS isolates including 22 different ST types to tilapia. The results (Figure 5) showed that ST23 strains, which have the broadest known host range in all *S. agalactiae* STs, are highly pathogenic to tilapia and also discovered that pathogenicity of CC19 strain to tilapia was closely related to its serotype. It is noteworthy that one ST103 strain and two ST930 strains (SLV of ST103) were highly pathogenic to tilapia, while 13 ST485 strains in the same genus CC103 were less or not pathogenic to tilapia. These results together with the recent increased isolation of tilapia ST103 strain and bovine ST103 strain in China and Brazil showed that ST103 strain could co-infect or cross infect humans, dairy cows and fish, while ST485 has evolved into a highly suitable human-specific strain (Godoy et al., 2013; Zhang et al., 2017). At the same time, the isolates of two human pathogenic ST-1 strains had opposite pathogenicity to tilapia. NNA048 is strongly pathogenic to tilapia, showing no difference from that of tilapia GBS, while NNA038 is not pathogenic to tilapia. Our further study (Wang et al., 2017) showed that that there was a 49.8 kb long, intact phage sequence encoding 68 proteins in NNA048 genome. All these results indicate that human ST103, ST23, ST1, and ST19 strains are highly possible to infect fish. Especially, ST103 strain has been isolated from tilapia (Godoy et al., 2013; Zhang et al., 2017). Compared to infection of fish with human GBS, epidemic spread of fish Streptococcus poses a greater threat to human health. In recent 20 years, there are many reports on human infection with fish Streptococcus (Weinstein et al., 1997; Lau et al., 2003; Koh et al., 2004). The study also confirmed that the current commercial fish implicitly carry a large number of *S. agalactiae* and eating fish was directly associated with human GBS infection (Foxman et al., 2007; Sun et al., 2016; Tan et al., 2016). Therefore, timely and effective prevention and control of the occurrence of fish Streptococci is very important to human health.

In conclusion, the findings of (1) high prevalence of CC103 GBS in human and cows, (2) formation of highly pathogenic, human specific infectious ST485 strain, (3) isolation of human CC67 strain that carries CC17 strain-specific HvgA gene, and (4) high pathogenicity of human ST23 strain to tilapia showed that there was co-infection or cross-host infection among human, bovine and fish GBS, and also provided new evidences for studying the evolutionary origin of highly pathogenic human CC17 strain.

## Author contributions

LL, RW, YH, TH, and FL: Contributed equally to this work; LL, RW, YH, TH, XY, and AL: Performed experiments and analyzed data; FL and WH: Provided software and bioinformatics expertise; MC and XG: Designed the experiments, analyzed data and wrote the manuscript.

### Conflict of interest statement

The authors declare that the research was conducted in the absence of any commercial or financial relationships that could be construed as a potential conflict of interest.
