# Supplementary material for: High Incidence of Pathogenic Streptococcus agalactiae ST485 Strain in Pregnant/Puerperal Women and Isolation of Hyper-Virulent Human CC67 Strain
Source: Front Microbiol. 2018 Feb 6;9:50. doi: 10.3389/fmicb.2018.00050 (PMC5808242; doi:10.3389/fmicb.2018.00050)
Supplement: Supplementary file 2 [file Table2.DOC]

**Table S2. Primers used in the study for amplification of pilus island (PI) and gbs2018 genes.**

| Target gene | Primer sequence |
| --- | --- |
| PI-1 |  |
| Forward | 5-CCGGGCTCATCCATGGGGACT-3 |
| Reverse | 5-ACAGCAGTCTCTGCCTGTCCGA-3 |
| PI-2a |  |
| Forward | 5-ATGGTTCGTACTTTGGTCGTGCTT-3 |
| Reverse | 5-TCGGCCTGTTTCAACTTTTCGCT-3 |
| PI-2b |  |
| Forward | 5-ACCGGCTGAAGGTATTGTTGCGA-3 |
| Reverse | 5-GCCACCATACTTGTCCAGTAAACGG-3 |
| gbs2018-A, gbs2018-B |  |
| Forward | 5-AAAATAAACGTGGTCCTATCCT -3 |
| Reverse | 5-CTTGAGAACGTCTTGACTGC-3 |
| gbs2018-B |  |
| Forward | 5-AGCACAGGAAGTTGCCCAGAAA-3 |
| Reverse | 5-AGCATCACGTAGCTTGTTAG-3 |
| gbs2018-C |  |
| Forward | 5-GTTGACCAAGCTTATGATCATGTGG-3 |
| Reverse | 5-TTAAATCCTTCCTGACCATTCC-3 |
