# Supplementary material for: High Incidence of Pathogenic Streptococcus agalactiae ST485 Strain in Pregnant/Puerperal Women and Isolation of Hyper-Virulent Human CC67 Strain
Source: Front Microbiol. 2018 Feb 6;9:50. doi: 10.3389/fmicb.2018.00050 (PMC5808242; doi:10.3389/fmicb.2018.00050)
Supplement: Supplementary file 5 [file Table5.DOC]

**Table S5. Correlation analysis of the disease source of isolates and its pilus island (PI) type.**

| Disease of patient | PI-2a | | | PI-2b | | | PI-1+PI-2a | | | PI-1+PI-2b | | |
| --- | --- | --- | --- | --- | --- | --- | --- | --- | --- | --- | --- | --- |
|  | DC,NO. | OC,NO. | P Value | DC,NO. | OC,NO. | P Value | DC,NO. | OC,NO. | P Value | DC,NO. | OC,NO. | P Value |
| Vaginitis | 6/17(35%) | 15/75(20%) | 0.15 | 6/30(20%) | 15/62(24%) | 0.434 | 8/43(17%) | 13/49(27%) | 0.257 | 1/2(50%) | 20/90(22%) | 0.406 |
| Threatened abortion | 0/17(0%) | 15/75(20%) | 0.035* | 6/30(20%) | 9/62(15%) | 0.350 | 8/43(17%) | 7/49(14%) | 0.39 | 1/2(50%) | 14/90(16%) | 0.301 |
| Premature rupture of membranes | 1/17(6%) | 13/75(17%) | 0.215 | 4/30(13%) | 10/62(16%) | 0.494 | 9/43(21%) | 5/49(10%) | 0.128 | 0/2(0%) | 14/90(16%) | 0.717 |
| Cervicitis | 1/17(6%) | 8/75(11%) | 0.475 | 4/30(13%) | 5/62(8%) | 0.326 | 4/43(9%) | 5/49(10%) | 0.583 | 0/2(0%) | 9/90(10%) | 0.813 |
| Urethritis | 5/17(29%) | 3/75(4%) | 0.005* | 1/30(3%) | 7/62(11%) | 0.195 | 2/43(5%) | 6/49(12%) | 0.18 | 0/2(0%) | 8/90(9%) | 0.833 |
| Pelvic inflammatory disease | 2/17(12%) | 4/75(5%) | 0.306 | 1/30(3%) | 5/62(8%) | 0.358 | 3/43(7%) | 3/49(6%) | 0.597 | 0/2(0%) | 6/90(7%) | 0.873 |
| EOD | 0/17(0%) | 4/75(5%) | 0.435 | 4/30(13%) | 0/62(0%) | 0.010* | 0/43(0%) | 4/49(8%) | 0.076 | 0/2(0%) | 4/90(4%) | 0.914 |
| LOD | 0/17(0%) | 2/75(3%) | 0.663 | 2/30(7%) | 0/62(0%) | 0.048* | 0/43(0%) | 2/49(4%) | 0.281 | 0/2(0%) | 2/90(2%) | 0.957 |
| Bronchopneumonia | 0/17(0%) | 2/75(3%) | 0.663 | 0/30(0%) | 2/62(3%) | 0.452 | 2/43(5%) | 0/49(0%) | 0.216 | 0/2(0%) | 2/90(2%) | 0.957 |
| Inevitable abortion | 0/17(0%) | 1/75(1%) | 0.815 | 0/30(0%) | 1/62(2%) | 0.674 | 1/43(2%) | 0/49(0%) | 0.467 | 0/2(0%) | 1/90(1%) | 0.978 |
| Premature delivery | 0/17(0%) | 1/75(1%) | 0.815 | 0/30(0%) | 1/62(2%) | 0.674 | 1/43(2%) | 0/49(0%) | 0.467 | 0/2(0%) | 1/90(1%) | 0.978 |
| Late production | 1/17(6%) | 0/75(0%) | 0.185 | 0/30(0%) | 1/62(2%) | 0.674 | 0/43(0%) | 1/49(2%) | 0.533 | 0/2(0%) | 1/90(1%) | 0.978 |
| Ectopic pregnancy | 1/17(6%) | 0/75(0%) | 0.185 | 0/30(0%) | 1/62(2%) | 0.674 | 0/43(0%) | 1/49(2%) | 0.533 | 0/2(0%) | 1/90(1%) | 0.978 |
| Infection of cesarean section | 0/17(0%) | 1/75(1%) | 0.815 | 0/30(0%) | 1/62(2%) | 0.674 | 1/43(2%) | 0/49(0%) | 0.467 | 0/2(0%) | 1/90(1%) | 0.978 |
| Chronic suppurative sinusitis | 0/17(0%) | 1/75(1%) | 0.815 | 1/30(3%) | 0/62(0%) | 0.326 | 0/43(%) | 1/49(2%) | 0.533 | 0/2(0%) | 1/90(1%) | 0.978 |
| Diabetes mellitus | 0/17(0%) | 1/75(1%) | 0.815 | 0/30(0%) | 1/62(2%) | 0.674 | 1/43(2%) | 0/49(0%) | 0.467 | 0/2(0%) | 1/90(1%) | 0.978 |
| Cardiovascular disease | 0/17(0%) | 1/75(1%) | 0.815 | 0/30(0%) | 1/62(2%) | 0.674 | 1/43(2%) | 0/49(0%) | 0.467 | 0/2(0%) | 1/90(1%) | 0.978 |
| Male infertility | 0/17(0%) | 1/75(1%) | 0.815 | 1/30(3%) | 0/62(0%) | 0.326 | 0/43(0%) | 1/49(2%) | 0.533 | 0/2(0%) | 1/90(1%) | 0.978 |
| Dermatosis | 0/17(0%) | 1/75(1%) | 0.815 | 0/30(0%) | 1/62(2%) | 0.674 | 1/43(2%) | 0/49(0%) | 0.467 | 0/2(0%) | 1/90(1%) | 0.978 |
| Fever | 0/17(0%) | 1/75(1%) | 0.815 | 0/30(0%) | 1/62(2%) | 0.674 | 1/43(2%) | 0/49(0%) | 0.467 | 0/2(0%) | 1/90(1%) | 0.978 |

Abbreviations: DC, disease of patient, OC, all other diseases.

* P < .05 compared with OC.
