# Supplementary material for: High Incidence of Pathogenic Streptococcus agalactiae ST485 Strain in Pregnant/Puerperal Women and Isolation of Hyper-Virulent Human CC67 Strain
Source: Front Microbiol. 2018 Feb 6;9:50. doi: 10.3389/fmicb.2018.00050 (PMC5808242; doi:10.3389/fmicb.2018.00050)
Supplement: Supplementary file 6 [file Table6.DOC]

**Table S6. Correlation analysis of the disease source of isolates and its gbs2018 gene type.**

| Disease of patient | gbs2018-A | | | gbs2018-B | | | gbs2018-C | | |
| --- | --- | --- | --- | --- | --- | --- | --- | --- | --- |
|  | DC,NO. | OC,NO. | P Value | DC,NO. | OC,NO. | P Value | DC,NO. | OC,NO. | P Value |
| Vaginitis | 8/38(21%) | 13/54(24%) | 0.468 | 12/41(29%) | 9/51(18%) | 0.142 | 1/13(8%) | 20/79(25%) | 0.146 |
| Threatened abortion | 5/38(13%) | 10/54(19%) | 0.349 | 9/41(22%) | 6/51(12%) | 0.151 | 1/13(8%) | 14/79(18%) | 0.329 |
| Premature rupture of membranes | 7/38(18%) | 7/54(13%) | 0.333 | 5/41(12%) | 9/51(18%) | 0.336 | 2/13(15%) | 12/79(15%) | 0.629 |
| Cervicitis | 4/38(11%) | 5/54(9%) | 0.554 | 4/41(10%) | 5/51(10%) | 0.638 | 1/13(8%) | 8/79(10%) | 0.627 |
| Urethritis | 6/38(16%) | 2/54(4%) | 0.05* | 1/41(2%) | 7/51(14%) | 0.058 | 1/13(8%) | 7/79(9%) | 0.685 |
| Pelvic inflammatory disease | 4/38(11%) | 2/54(4%) | 0.19 | 1/41(2%) | 5/51(10%) | 0.16 | 1/13(8%) | 5/79(6%) | 0.61 |
| EOD | 0/38(0%) | 4/54(7%) | 0.113 | 1/41(2%) | 3/51(6%) | 0.395 | 3/13(23%) | 1/79(1%) | 0.008* |
| LOD | 0/38(0%) | 2/54(4%) | 0.342 | 0/41(0%) | 2/51(4%) | 0.305 | 2/13(15%) | 0/79(0%) | 0.019* |
| Bronchopneumonia | 0/38(0%) | 2/54(4%) | 0.342 | 2/41(5%) | 0/51(0%) | 0.196 | 0/13(0%) | 2/79(3%) | 0.736 |
| Inevitable abortion | 1/38(3%) | 0/54(0%) | 0.413 | 0/41(0%) | 1/51(2%) | 0.554 | 0/13(0%) | 1/79(1%) | 0.859 |
| Premature delivery | 0/38(0%) | 1/54(2%) | 0.587 | 1/41(2%) | 0/51(0%) | 0.446 | 0/13(0%) | 1/79(1%) | 0.859 |
| Late production | 0/38(0%) | 1/54(2%) | 0.587 | 1/41(2%) | 0/51(0%) | 0.446 | 0/13(0%) | 1/79(1%) | 0.859 |
| Ectopic pregnancy | 0/38(0%) | 1/54(2%) | 0.587 | 1/41(2%) | 0/51(0%) | 0.446 | 0/13(0%) | 1/79(1%) | 0.859 |
| Infection of cesarean section | 1/38(3%) | 0/54(0%) | 0.413 | 0/41(0%) | 1/51(2%) | 0.554 | 0/13(0%) | 1/79(1%) | 0.859 |
| Chronic suppurative sinusitis | 0/38(0%) | 1/54(2%) | 0.587 | 1/41(2%) | 0/51(0%) | 0.446 | 0/13(0%) | 1/79(1%) | 0.859 |
| Diabetes mellitus | 0/38(0%) | 1/54(2%) | 0.587 | 1/41(2%) | 0/51(0%) | 0.446 | 0/13(0%) | 1/79(1%) | 0.859 |
| Cardiovascular disease | 1/38(3%) | 0/54(0%) | 0.413 | 0/41(0%) | 1/51(2%) | 0.554 | 0/13(0%) | 1/79(1%) | 0.859 |
| Male infertility | 0/38(0%) | 1/54(2%) | 0.587 | 0/41(0%) | 1/51(2%) | 0.554 | 1/13(8%) | 0/79(0%) | 0.141 |
| Dermatosis | 1/38(3%) | 0/54(0%) | 0.413 | 0/41(0%) | 1/51(2%) | 0.554 | 0/13(0%) | 1/79(1%) | 0.859 |
| Fever | 0/38(0%) | 1/54(2%) | 0.587 | 1/41(2%) | 0/51(0%) | 0.446 | 0/13(0%) | 1/79(1%) | 0.859 |

Abbreviations: DC, disease of patient, OC, all other diseases.

* P < .05 compared with OC.
